# Community Water Fluoridation a Cost–Benefit–Risk Consideration

**DOI:** 10.1002/puh2.70009

**Published:** 2024-11-27

**Authors:** Bill Osmunson, Griffin Cole

**Affiliations:** ^1^ Private Practice Bellevue Washington USA; ^2^ Center for Advanced Dental Disciplines (CADD) Fort Collins Colorado USA; ^3^ American College of Integrative Biological Dental Medicine Saddlebrook New Jersey USA; ^4^ International Academy of Oral Medicine and Toxicology Champions Gate Florida USA

**Keywords:** dental fluorosis, developmental neurotoxicity, fluoridation, IQ

## Abstract

For over 70 years, the addition of fluoride to public water as community water fluoridation (CWF or fluoridation) with intent to prevent dental caries continues to be controversial, and risks are seldom included in monetary evaluations. Published operational costs and benefits of fluoridation are used, whereas published and clinical experience treating dental fluorosis are utilized to estimate treatment costs of patient‐perceived dental fluorosis and lost wages from lower IQ (intelligence quotient). Published estimated caries averted, less operational costs at $8 PPPY (per person per year) were used, compensation for functional and cosmetic dental fluorosis $126 PPPY, and lower earnings from presumed harm of developmental neurotoxicity estimated at $438 PPPY. Net loss from CWF is estimated at $556 PPPY, although some individuals will have significantly more or less loss. Previous economic evaluations of fluoridation have estimated caries averted and costs of operations; however, few evaluations include the costs of treating harm. Fluoridation is not cost‐effective if the cost of harm is included. Alternatives for the prevention of dental caries should be promoted, and the cessation of fluoridation is indicated.

## 1 Introduction

Community water fluoridation (CWF) or “fluoridation” is referred to here as the addition of fluoride into public water by authorities with the singular intent to “prevent or mitigate” dental caries, a disease. Estimates of about 400 million people world‐wide have fluoride added to their drinking water, with more than half of those in the United States of America (USA). In contrast, about 97% of Europe is without CWF and has a similar prevalence of dental caries. Endemic fluoride, which is a major health problem in over 20 countries, is not the direct focus of this analysis. Fluoridation is controversial [1].

Topical fluoride in toothpaste is approved by the US Food and Drug Administration Center for Drug Evaluation and Research with a New Drug Approval (FDA CDER NDA) as a drug (medicine) with label and dosage. The label is precise and clear: use a “pea”, “rice”, or “smear” size that contains about a quarter milligram of fluoride and the warning, “do not swallow.” Incidentally, this contains the same dosage as a glass of fluoridated water. Acute toxicity of fluoride is estimated at 5 mg/kg body weight. In contrast, neither fluoridated water nor fluoride supplements (pills or liquids) have FDACDERNDA approval and are considered unapproved prescription drugs, misbranded, illegal, and adulterated (Supporting Informations A, B, and E).

Dental caries is not a fluoride deficiency disease. Fluoride is not an essential nutrient because a lack of fluoride does not cause dental caries or any disease. Many public health agencies in the USA claim every dollar spent on fluoridation saves $38 in dental treatment [2] and assume negligible harm. However, the public has concerns with the safety of fluoride [3], and adding fluoride to tap water may not be effective, in part, because dental caries have significantly declined with or without fluoridation [4]. A national survey [9] reported about 60% of children and adolescents have dental fluorosis, a biomarker of excess fluoride ingestion prior to 8 years of age.

A single clinician's mistake may harm the patient being treated and result in compensation for that patient. Of far greater tragedy, especially for low socioeconomic populations, is a well‐intended public health policy mistake, potentially harming millions, ‐while lacking individual freedom of consent, and inability to be compensated for the harm. As public health authorities, we must ensure the public water is safe for all, because water is essential for life. Prior economic evaluations of CWF [22] have almost always omitted potential harm, considered the harm to be negligible, or discounted the harm as a “side‐effect.” Many in the public have concerns for both safety [3] and cosmetics [11].

Potential harms were reported by the National Research Council in 2006 [5] to such structures and physiologic functions as cell function, teeth, skeleton, chondrocyte metabolism, arthritis, reproductive and developmental effects, neurotoxicity, neurobehavioral effects, endocrine system, gastrointestinal, renal, hepatic, immune systems, genotoxicity and carcinogenicity. More recently, concerns of potential low birth weight, miscarriage, and increased infant mortality have been raised. Safety should be assured by authorities rather than patients being required to prove that the policy is flawed. Randomized controlled trials, required for FDA CDER approval, safety, dosage, label, and individual consent are lacking. For this reason, we aimed this analysis to incorporate into previous estimates of CWF costs (using 2021 USA dollars) of two adverse effects: dental fluorosis and lower income due to developmental neurotoxicity (Supporting Information E, Assumptions).

To develop this article, we reviewed publications on Medline and Google Scholar for costs, benefits, and risks of fluoridation that resulted in thousands of hits. A search with PubMed of “cost of water fluoridation harm” resulted in 1 report from 1982 and Google Scholar that resulted in thousands of hits. The majority of the literature suggests fluoridation is environmentally sustainable and effective, and the risk to the individual is negligible, perhaps a side effect, but not specifically harmful [6].

## 2 Costs

Published estimated costs per person per year (PPPY) to fluoridate water and estimated caries treatment costs based on clinical experience and insurance reimbursements are used. Costs to treat dental fluorosis and lower wages from lower intelligence quotients (IQs) are estimated based on published and clinical experience.

Discount rates generally vary from 0% to 5%. In this analysis, a discount rate of 0% was chosen for two reasons. First, this evaluation considers lifetime harm rather than the potential benefit. Second, the assumption is made that patients expect full compensation. Pain, suffering, time, adverse effects, and treatment failure should be considered but are not included.

## 3 Uncertainty

The degree of certainty that ingested fluoride during tooth development causes dental fluorosis is high with strong consensus. The cost for treating dental fluorosis has less certainty as dental fluorosis is often brushed aside as only “cosmetic” and therefore negligible and functional harm is ignored. The effects of low fluoride exposure on developmental neurotoxicity are rapidly developing and presumed, although less certain.

### 3.1 CWF Operating Costs and Benefits

A MEDLINE review provided a range of net benefit for fluoridation generally from $8 PPPY [2] to $41 PPPY, and costs of treating harm are omitted or considered negligible. When costs are mentioned, not all costs are includedsuch as accidental spills and transportation accidents, overfeeds, infrastructure installation and maintenance, costs to avoid fluoridation, land issues, reasonable employee wages, benefits, training, supplies, research promoting and opposing fluoridation, defending against challenges to fluoridation, and costs to treat and compensate for adverse health effects. If observational studies are dismissed, evidence of fluoridation benefit or risk would be lacking (Supporting Information C). Randomized controlled trials of fluoridation are lacking.

The main body of evidence for potential dental caries mitigation has numerous limitations and assumptions (Supporting Information C), yet the body of published evidence suggests fluoridation may have had and continues to have some mitigating effect on dental caries at 1 ppm for children [6, 7, 8].

### 3.2 Dental Fluorosis

Fluoridation is authority‐controlled but not individual dosage or total individual exposure‐controlled, because not everyone drinks the same amount of water or has the same amount of fluoride intake from other sources.

A US Environmental Protection Agency funded study [10] (1987), with fluoride concentrations between 1.0 and 4.0 mg/L, evaluated the cost of treating dental fluorosisfinding:

“A mean cost for all consultants shows that the estimated costs for restoring function exceeds the cosmetic costs in all categories except the minimum later costs. This represents a new finding and raises an issue that has been overlooked or ignored by previous investigators and the profession, i.e. that repair of the cosmetic discoloration was the only cost involved; or that repair of dysfunction was never considered to be a problem.”

All consultants do not appear to have been cosmetic dentists nor did they estimate lifetime costs. Dental insurance companies usually reject payment for the initial cosmetic treatment and consider wear as a “natural‐phenomena” usually not covered. “Damage is the cost, not the repair.” Patient #1 (below, used with patient consent) has a normal ideal smile with healthy teeth, no fluorosis detected, and was raised predominantly on mother's milk, and no formula was made with fluoridated water.

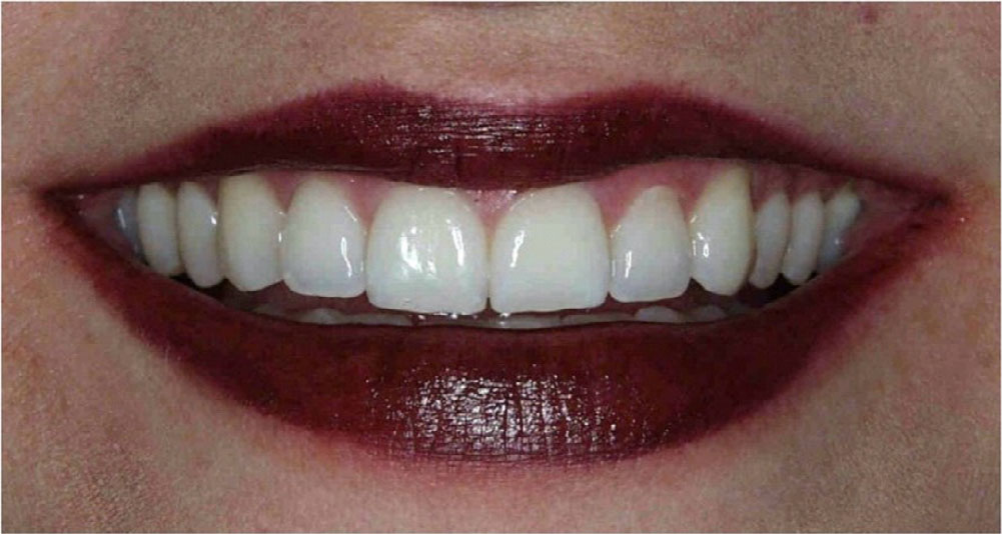

For comparison, Patient #2 (below) was diagnosed with Dean's Fluorosis Index of 4, “discrete or confluent pitting,” moderate to severe dental fluorosis and has functional damage with chipped, pitted, and worn teeth.

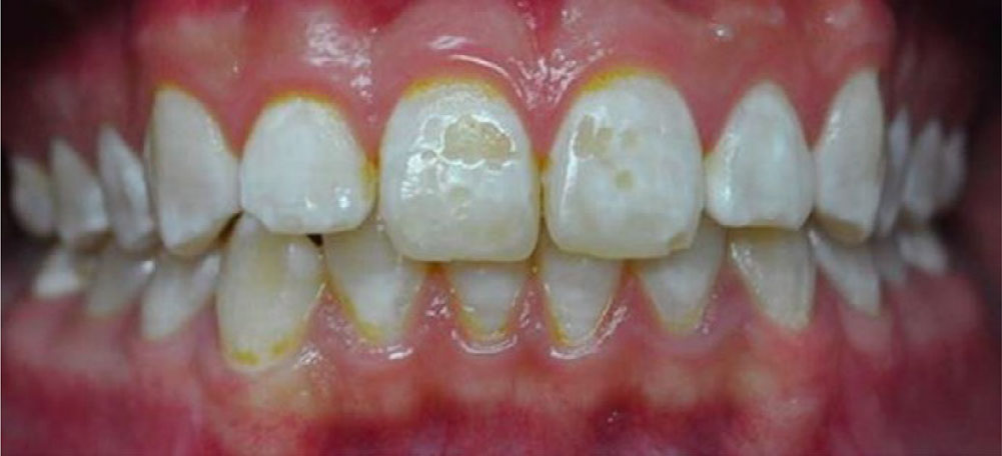

Patient #2 (used with patient consent) was raised mostly on a formula made with fluoridated water. Mom was confident that fluoridated toothpaste was not swallowed and no fluoride supplements were ingested.

The diagnostic classification of fluorosis has some value to the researcher but seldom to the patient. For patient #2, the top two front teeth are the most damaged; however, restoring just those two teeth would emphasize the adjacent moderate and mild fluorosis. Each patient and  his/her dentist decide how many teeth have been damaged and should or should not be treated. In this case, 24 teeth had cosmetic and functional dental fluorosis damage.

Professional diagnosis of dental fluorosis is commonly reported at 14.5%–17.5% [11, 12]. However, 52% of patients perceived their dental fluorosis, at 0.7 mg/L fluoride in CWF, to be objectionable, and 95% of those wanted the damage repaired [12].

This analysis uses a conservative 30% of those on fluoridation will have perceived dental fluorosis they would wish to have removed or for which they would request compensation. Options for treatment vary depending on the extent of damage, patient preference, and treating clinician.

The options are divided here into two general groups. An estimated 20% of those on fluoridated water would choose a conservative “Option A” and 10%, a comprehensive “Option B.” Costs for treating functional damage have less documentation and are not specifically estimated here, although functional damage may cost more than cosmetic damage.

## 4 Costs to Treat Cosmetic Dental Fluorosis

The EPA study [10] of professional diagnosis rather than patient perception appears to have assumed optimum care would last a lifetime and reported a range between $660 and $12,000 per patient (all costs in USA dollars converted to 2021 value). Dental fluorosis may be managed by bleaching, micro‐abrasion, resin infiltration, veneering, or full coverage of the tooth such as crowns.

### 4.1 Clinical Treatment Option A

Micro‐abrasion grinding of the outer layer of enamel, sealants, or resin infiltration (fillings) can improve dental fluorosis appearance and minor functional damage. Bleaching of teeth prior to resin infiltration can improve cosmetics but tends to whiten all areas, and a contrast in shade is not always considered fully restored. Repeated treatment or “touch up” bleaching and/or minor restorations and re‐treatments are estimated every 5 years for a conservative $100 PPPY for 60 years. Depending on case presentation, an estimated 20% of those exposed to CWF with perceived dental fluorosis and minor functional damage would accept a bleaching/micro‐abrasion and/or minor filling treatment. A general inflation rate of 3.57% and inflation of dental fees of 4.33% is used (Supporting Information E Tables S1 and S2). Patients requesting compensation for harm would expect full compensation, and a zero‐discount rate is used.

#### 4.1.1 Summary of Micro‐Abrasion Option A

Clinically based estimated cost of $100 a year per person × 60 years = $6000. An additional $1200 for the difference of general inflation at 3.57% and dental inflation at 4.33% for $7200. An estimated 20% of the population on CWF is assumed to choose Option A that is $1440 × 1.46% of the population at each age = $21 PPPY (Supporting Information E Table S1).

#### 4.1.2 Comprehensive Clinical Treatment Option B

If offered compensation for the damage, many would choose the highest quality of treatment. Comprehensive cosmetic and functional treatments are estimated based on experience and dental insurance fees at $1200 per tooth. Classification of dental fluorosis is based on the two worst teeth, although 1–32 teeth can be damaged. If costs are not the controlling factor, a cosmetic patient will want several or all upper and lower teeth treated inclusive of functional damage. An estimate is used here for an average of 10 teeth per person at $1200 of damage per tooth. This estimate is at the high end of the EPA study [10] and yet in keeping with another published study on the treatment of dental fluorosis harm [2] and insurance payments. The original treatment is estimated to be replaced an average of four times during a person's life. Re‐treatment often progresses to more complex pathology and more extensive treatment. These additional expenses are not included here. A dental school clinic reported “average survival time” for all crowns was 4.4 years. Reports of restoration survival at 95% at 10 years and 75% at 18 years are consistent with clinical experience. Dental insurance companies seldom pay for re‐treatment of crowns within 5 years. A 12‐year survival expectancy for high‐quality treatment is used here.

#### 4.1.3 Summary of Option B

$1200 × 10 teeth = $12,000 × 5 treatments = $60,000 add $12,000 (Supporting Information E, Table S2) for a difference in inflation rates = $72,000 × 10% on fluoridation choosing optimal treatment = $7200 × 1.46% of the population at each age = $105 PPPY. Combining Options A of $21 PPPY with Option B of $105 PPPY equals a conservative estimate of $126 PPPY for the treatment of dental fluorosis.

#### 4.1.4 Developmental Neurotoxicity

(Supporting Information D) Fluoride was nominated to the USA National Toxicology Program (NTP) in 2015 for review of fluoride's possible neurodevelopment and cognitive health effects and was accepted. Over 8 years and several peer reviews later, the Board of Scientific Councilors approved a draft. The current NTP draft review included 159 human studies, 339 non‐human studies, 60 in vitro, and many other publications, and 95% of the highest quality studies reported lower IQ. The original draft reported a presumed neurodevelopment hazard. The draft monogram was blocked from release until the court [13] ordered release; although the report has still not been published (Supporting Information F). The report [14] has been divided into two sections, one called the “state of the science” and the second the “meta‐analysis.” Reviewers of the NTP monograph have generally focused on clarity and strengthening the report rather than disagreement with the conclusion. Some reviewers suggested *“that the monograph is not designed to be informative regarding decisions about fluoride concentrations for water fluoridation.”*[14] The NTP authors disagreed, confirming they were considering total exposure, not just fluoride from fluoridation. Evaluations of drugs/medicines are not in the jurisdiction of the NTP, and fluoridated water is only one source of fluoride exposure rather than an individual dosage. In addition, water consumption is not controlled and averages about 1 L/day; however, water consumption can exceed 10 L/day [5].

Subsequent to the NTP cut‐off date, research has remained reasonably consistent, a *“0.5 mg increase in fluoride intake from infant formula corresponded to an 8.8‐point decrement in Performance IQ” and a “4.4 FSIQ (Full Scale IQ) points among preschool children who were formula‐fed in the first six months of life for each 0.5 mg/L increase in water fluoride concentration* [16].” A prospective study reported, *“a 0.68 mg/L (i.e., 1 IQR) increase in specific gravity–adjusted maternal urinary fluoride during pregnancy was associated with nearly double the odds of T scores for total child neurobehavioral problems being in the borderline clinical or clinical range*. ***Meaning***: *These findings suggest that prenatal fluoride exposure may increase risk of neurobehavioral problems among children living in an optimally fluoridated area in the US* [25].”

The fluoride concentration set by authorities in fluoridating water is a concentration and not a dosage. An intraspecies uncertainty factor and margin of error of at least 10 should be used for differences in individual water consumption. Additional factors for individual health, diet, and cumulative and synergistic toxins should be considered.

Nine mother–child neurotoxicity studies have been published [17]. Eight of the studies (the first of USA/Canadian cohorts in 2020) reported neurotoxicity at low concentrations. One outlier [18] reported an increase of +15 IQ points per 1 mg/g increase in MUFcr (Mother's Urinary Fluoride Creatinine) for boys, not girls. This is an impossible boost to IQ that has never been found for any other chemical, nutrient, or diet. Even less believable is the finding that in the non‐fluoridated zone where the water *F* concentration was 0.05 mg/L, the increase in IQ for those in the low fluoride communities was +28 IQ points per 1 mg/g MUFcr and when corrected for creatinine increased IQ to an impossible 38 IQ points. If true, most boys would be gifted geniuses, and fluoridated communities would have few if any boys in the special education classes. A delay in effect, huge increase for boys but not girls, and without supporting research indicates this study needs the laboratory work redone. The study is an outlier because it is the only human study out that has ever found a statistically significant benefit from the ingestion of fluoride.

Over 90% of the higher quality fluoride studies reported by the NTP at exposure levels of <1.5 mg/L fluoride in water or urine reported significant adverse neurotoxic effects [19]. A recent meta‐analysis of eight studies from non‐endemic fluoride communities [20] reported no effect on IQ scores. The study has limitations, such as including the outlier mentioned above.

There is reasonable agreement and consistency that fluoride is a developmental neurotoxin. This study is consistent with the NTP report and the majority of published research using a conservative 3 IQ loss for those on fluoridated water.

#### 4.1.5 IQ and Wages

A search at scholar.google of “IQ effect on wages,” resulted in 59,800 articles. Although lacking consensus, 1 IQ point increase appears to predict about 1% higher wages, about $500/IQ point [21], at the population level rather than individual. A lifetime income effect of 2.0% per IQ, or about $1000/year/IQ point, appears to be well supported [15]. We have chosen a conservative $500 PPPY.

### 4.2 Lower Wages Results

Summary: 3 IQ loss × $500/year/IQ loss = $1500 lower income/year. A total of 40 work years × $1500 lower income/year = $60,000 and assuming only 50% drink a significant amount of the CWF = $30,000 × 1.46% of the population at each age = $438 PPPY lower wages (Supporting Information E, Table S2).

## 5 Discussion

Dental fluorosis is a known risk of excess fluoride ingestion prior to 6−8 years of age. Given the cost of treating cosmetic dental fluorosis and lower wages due to lower IQ, fluoridation is not cost‐effective. When reviewing the populations at large, fluoride exposure appears dose‐related. However, some children get severe dental fluorosis with apparently low fluoride concentrations, and diet, genetics, elevation, chemical sensitivity, other toxins such as lead, and kidney function can be confounders. Some have suggested mild fluorosis is not a cosmetic concern. However, clinicians placing unsightly black mercury fillings and bright gold crowns may not have been cosmetically as sensitive to our patient's opinions. As an analogy, a scratch on a car is undisputed cosmetic damage. If patients find dental fluorosis has harmed their smile, those in authority contributing to or causing the damage must respect the patient's opinion. A National Productivity (Gross Domestic Productivity) and national IQ (the “Hive Mind”) affecting savings, cooperation, high‐value technologies, and market‐oriented policies appear to have a greater impact than individual IQ on individual wages. A negative effect on the “Hive Mind” is not included here, nor risks to bones, thyroid, mitochondria in cells, endocrine system, cancer, kidneys, and other risks from excess fluoride exposure. A benchmark dose analysis to consider a safe fluoride exposure reported about 0.2 mg/L maternal urine‐fluoride exposure (similar concentration for water) would be considered to date as safe [22].

## 6 Alternatives to Fluoridation

Comparing five preventive procedures, toothbrushing with toothpaste was the most effective preventive procedure, followed by fluoride varnish. Fluoridated water along with dental sealants and initial exams are considered less effective. Additionally, individual health education [23] with oral hygiene and nutrition would not only reduce dental caries but also periodontal disease and other health risks. Fluoride‐free toothpaste, such as biomimetic hydroxyapatite, should be considered [24, 25]. Money from a tax on carbonated beverages could be used for preventive education.

## 7 Conclusion

After almost 80 years of adding fluoride to community water, the controversy continues due to the current alleged efficacy. Lack of individual choice, ‐, risks, desired dosage, total exposure, jurisdiction, research quality, environmental justice, ethics, alternatives, and lack of a cost–benefit should all be included and considered. All streams of evidence should be considered for policy evaluation.

## Author Contributions

**Bill Osmunson**: conceptualization; methodology; formal analysis; supervision; funding acquisition; investigation; writing—original draft; writing—review and editing; project administration; resources; data curation. **Griffin Cole**: validation; writing—review and editing; funding acquisition; visualization.

## Conflicts of Interest

The authors declare no conflicts of interest.

## Supporting information



Supporting Information

## Data Availability

The data that support the findings of this study are available from the corresponding author upon reasonable request.
