# Supplementary material for: Community Water Fluoridation a Cost–Benefit–Risk Consideration
Source: Public Health Chall. 2024 Nov 27;3(4):e70009. doi: 10.1002/puh2.70009 (PMC12039709; doi:10.1002/puh2.70009)
Supplement: Supplementary file 1 — Supporting Information [file PUH2-3-e70009-s001.docx]

Community Water Fluoridation: A Cost-Benefit-Risk Consideration

**SUPPLEMENT A: JURISDICTION**

The FDA has jurisdiction over the dosage, label, efficacy and safety of fluoride when the intent of use is to prevent, mitigate or treat human disease. No exemption is made for dilution in water.

Fluoride is defined in laws as a poison or highly toxic substance and exempt from those laws when regulated as a drug or pesticide, but not exempt as a food or mineral. The absence of fluoride in the diet does not cause dental caries and fluoride is not an essential nutrient.

Fluoride is in the legal definition of a drug when used with the intent to prevent disease, the key is “intent.” In all countries considered, jurisdiction over substances used with the intent to prevent disease, such as fluoride, are under drug or medicine regulatory jurisdictions.

The FDA testified to Congress that fluoride is a drug. Fluoride toothpaste is approved and has a drug label with warning, “Do Not Swallow,” referring to a pea size amount, 0.25 mg of fluoride, the same amount of fluoride in a glass of fluoridated water at 0.7 ppm (mg/l).

The EPA’s legal-council regarding CWF advised, *“the FDA, remains responsible for regulating the addition of drugs to the water supply for health care purposes.”*

The Safe Drinking Water Act includes*, “No national primary drinking water regulation may require the addition of any substance for preventive health care purposes unrelated to contamination of drinking water.”* Congress has prohibited the EPA from adding anything to water which has an intent to prevent disease. The individual amount of water consumed is not controlled with a range of little or no tap water to over 10 liters a day, and thus the dosage of fluoride consumption is uncontrolled.

Fluoride is regulated by the U.S. EPA as a contaminant in drinking water and as an air pollutant.

About 97% of Europe is CWF free along with Israel, China and Japan. CWF is without label, misbranded, and adulterated failing to conform as a drug to compendium standards of purity.

IM (Institute of Medicine) repots infant formula made with water , *“can range from as little as 0.1 to over 1.0 mg/day of fluoride.”*

Current scientific evidence supports the USA Environmental Protection Agency (EPA) scientists’ statement in 2001:

***“In summary, we hold that fluoridation is an unreasonable risk. That is, the toxicity of fluoride is so great and the purported benefits associated with it are so small - if there are any at all – that requiring every man, woman and child in America to ingest it borders on criminal behavior on the part of governments.”***

Sodium fluoride is listed as a drug in the Pharmacopeias. In 1975, Drug Digest reported the FDA notified 35 fluoride manufacturers of fluoride supplements (pills or liquid drops) there is no substantial evidence of drug effectiveness and marketing is in violation of the new drug provisions. Again in 2016, the Seattle office of the FDA sent a warning letter to a fluoride supplement manufacturer that their fluoride products were unapproved, misbranded, not generally recognized as safe and effective. No FDA-approved applications were on file for the fluoride drugs as listed.

Two USA state Boards of Pharmacy have determined fluoride ingestion with intent to prevent disease is a drug. Topical fluoride is not listed in the FDA Orange Book of approved drugs.

**SUPPLEMENT B: BOTTLED WATER**

The FDA was “notified” under section 403(r)(3)(C) § (21 U.S.C. 343(r)(3)(C) of the Federal Food, Drug, and Cosmetic Act that a health claim would be made for fluoride added to bottled water. The health claim did not go through the drug approval process. No Randomized Controlled Trials were provided for efficacy, safety or dosage. The recommendation was made based on authoritative statements of the CDC, Surgeon General and Public Health Service. The Surgeon General oversees the Public Health Service. Neither the Surgeon General nor CDC have drug regulatory authority.

The FDA did not have the option to deny the notification; however, the FDA requires companies adding fluoride to bottled water to add to the label a warning the water is not to be used for children under two years of age, 21 CFR 101.14(e)(5). The health claim is not intended for use on bottled water products specifically marketed for use by infants. Current developmental neurotoxicity studies do not support an exemption of CWF from the same warning.

The 2006 NRC report on fluoride in drinking water and EPA 2010 Relative Source Contribution report the mean individual adult intake of water is just under 1 liter/day or about 330 million liters or 87 million gallons/day, over 30 billion gallons/year. In 2020 about 15 billion gallons of bottled water were sold in the USA which did not contain fluoride. Research on CWF has not usually factored in consumer shift to bottled water, making determination of caries averted and risks more complex. And the shift puts a burden on those with lower incomes.

Questions should be considered. Does bottled water low in fluoride increase caries prevalence? The answer appears to be “no.” Are researchers controlling for bottled water use when evaluating dental caries rates? Most studies do not. The dosage of fluoride from all sources should be considered.

**SUPPLEMENT C: CARIES AVERTED**

- **No Known Mechanism**

Fluoride works by interacting topically after teeth erupt. The evidence for its effectiveness when applied to erupted teeth is well supported. Fluoride incorporation into developing teeth prior to eruption was previously thought to provide resistance to future dental decay but the levels are not high enough to provide significant contribution to caries prevention.

A very small amount of ingested fluoride makes its way to saliva to provide some topical fluoride after tooth eruption, but this amount is 50 to 100-fold less than what is obtained from fluoride naturally occurring in food and beverages. *“The enamel demonstrated significant transport hindrance for the ions, and the effective pore radii of the transport pathways in the enamel were found to be approximately 0.7-0.9 nm.”*

**2) No Randomized Controlled Trials (RCT)**

There have been no randomized, double blinded clinical trials published in any population of humans. The Cochrane Library reported one prenatal RCT fluoride supplement and found no statistical significant difference. The first RCT has started with fluoridated bottled water for infants.

Without a known mechanism coupled with lack of RCTs, the FDA is correct determining the evidence of benefit from fluoride ingestion is “incomplete.”

**3) Limited Confidence in Current CWF Efficacy Studies:**

Not one study has corrected for highly significant unknown confounding factors such as the highly significant unknown causing caries decline from about 11.5 cavities/12 year old in the USA during the 1930’s to about 5.5 cavities/12 year old before fluoridation, or the faster decline in non-CWF developed countries. Frequently studies lack control for: socioeconomic status, adequate size, diagnosis blinding, delay in tooth eruption, diet, salivary flow, use of fluoridated toothpaste or professional fluorides, and enamel hypoplasia. Total exposure, oral hygiene, life-time benefit, damage from excess fluoride, genetics, bias, or errors are also often not controlled. Neurath and later Cheng using WHO data demonstrate in developed countries, dental caries declined to similar low levels regardless of CWF or fluoridated salt in less time than the fluoridated countries. If equal and faster results have been achieved in caries reduction without fluoridation, a claim cannot be made that CWF is essential.

**4) No Known Effective Dosage**

Without RCT published studies required by the FDA, the individual dosage, mg/Kg/day, to mitigate dental caries has never been adequately determined. Concentration of fluoride in water is not dosage. Instead, an estimated Adequate Intake (AI) is used by the National Institute of Health. However, the implication of “adequate” is inadequately supported that fluoride has benefit, exposure has no upper limits, is without significant risk and below adequate intake causes dental caries.

For example, Komarek used data from time of tooth eruption and found no convincing effect of fluoride intake on caries development. Fluoride may cause a delay in eruption and caries development delayed rather than prevented.

Historical research suggested CWF was “remarkably effective,” however, current research is less confident. A major review in 2000 from the Centre for Reviews and Dissemination at the University of York (York Review) concluded that the best available evidence suggested that CWF reduced the prevalence of caries, and they reported the reduction was difficult to quantify from the evidence available. The authors also noted, “it is surprising to find that little high-quality research has been undertaken.”

The amount of fluoride ingested from CWF is not controlled. The average intake of water is estimated at just under 1 liter/day for adults, 90th percentile is just over 2 liters and some drink over 10 liters/day. To protect from potential harm a safety factor of 10 just for differences in fluoride intake should be used. Consideration for genetic sensitivity, general health, age, kidney function, pregnant women and infants on formula made with fluoridated water must be considered for policy review.

Evidence of excess fluoride intake is supported with rates of dental fluorosis increasing from about 10% to 70%, Current reports of moderate/severe between 7% to 28%, Espinoza raised concern with the quality of data which has Federal oversight and funding. However, Espinoza did not support the concern with the available unreleased photographic evidence for peer review.

Both the National Institute of Health and WHO recommend mother’s milk for infants as ideal. However, they do not reconcile the problem infants have on formula made with CWF ingesting an estimated 140 times more fluoride than breast fed babies. And Zohoori reported, *“In conclusion, a relatively large proportion of fluoride intake is retained in the body in weaned infants.”* CWF used to make infant formula places the infant at extreme risk.

**5) Lack of Label.**

Without label, consumers don’t know how much fluoride is in their foods such as mechanically deboned meat, tea, grapes, post-harvest fumigated foods with fluoride, etc. The only label is on fluoride toothpaste with a warning, “Do Not Swallow.” Bottled water with fluoride added is required to have a label.

**6) Systematic reviews of benefit**

A 2015 Cochrane systematic review looked at non-randomized, non-blinded before and after clinical 'experiments' on populations where only one community was examined in each 'experiment'. Too many variables were involved in the non-randomized, non-blinded trials they compared, such as controlling for diet, toothpaste use, and delayed tooth eruption. The Cochrane review combined early clinical trials before fluoridated toothpaste dominated the market with more recent trials and none looked at 0.7 ppm CWF.  Even lowering standards, the Cochrane systematic review was criticized for being too restrictive, suggesting Cochrane reviews primarily evaluate RCTs *“for new drugs and clinical interventions for use with individuals, not public health initiatives targeted at populations*.”

However, ethical reasoning would demand higher quality of evidence of benefit not lower quality for treatment without consent using an unapproved drug or under their doctor’s supervision. The Cochrane review raised concerns for lack of studies to determine; current benefit, lack of benefit for lower socioeconomic status, lack of risk with fluoridation cessation, 97% of studies at high risk of bias, substantial between-study variation, and no studies met their criteria to determine effectiveness for adults. Risks were not considered. The CDC also reported, “Ingestion of fluoride is not likely to reduce tooth decay.”

**SUPPLEMENT D: DEVELOPMENTAL NEUROTOXICITY**

The NTP Monograph (footnote 24 of paper) started in 2016 and first draft reviewed in 2019 and proposed a “hazard conclusion” according to the director’s declaration to the court, may never be published. The draft has been divided in the “State of the Science Monograph” and a Meta-Analysis Manuscript.”

NTP’s previous 21 monographs on other toxins were never subjected to a peer review by the National Academies of Science, Engineering, and Medicine (NASEM) let alone two peer reviews by NASEM (2020-2-21), a private non-transparent review by “various [HHS] entities” (2021-2022) including the Centers for Disease Control and the National Institute of Dental and Craniofacial Research, both strong promoters of CWF, and upcoming review by NTP’s Board of Scientific Counselors (BSC).

NASEM states: ***“NTP therefore should make it clear that the monograph cannot be used to draw any conclusions regarding low fluoride exposure concentrations, including those typically associated with drinking-water fluoridation.”***

The NASEM expects the NTP monograph to provide “a clear and convincing argument that supports its assessment” of developmental neurotoxicity but NASEM fails to provide the same clear and convincing argument to protect the fluoride exposure concentration typically associated with drinking water fluoridation.

However, the NASEM statement attempting to protect “drinking-water fluoridation” (CWF) lacks support for several reasons:

1. Concentration in water is not patient dosage. If NASEM had used milligrams of fluoride per kilogram of body weight per day the statement would have had greater support. The NASEM appears to assume everyone drinks the “mean” amount of water, 1 liter/day. In reality, some drink none and some drink over 10 liters/day. (NRC 2006)6 Concentration is controlled but dosage is not.

2. The NASEM correctly coaches NTP towards “a clear and convincing argument that supports its assessment.” In contrast, the NASEM makes a conclusion without evidence or clear and convincing argument to support the NASEM’s assessment that 0.7 ppm fluoride in public water is apparently safe.

3. The NASEM appears to assume public water is the only significant source of fluoride and/or at least all sources with the addition of CWF will be safe.

4. The NASEM assumes no margin of error, uncertainty factor, individual sensitivity, compromised health status or synergistic effects of other toxins. EPA uses a 1:1 uncertainty factor, absolute certainty 4 ppm fluoride (MCL) in public water is safe for at least 90% of the public and NASEM appears to have the same opinion.

MECHANISM OF FLUORIDE’S DEVELOPMENTAL NEUROTOXICITY

The mechanism of fluoride’s developmental neurotoxicity has been reported by the NTP with Han, and Goodman reporting low iodine intake may increase the risk from fluoride.

META-ANALYSIS

Choi (2012) used twenty-seven of the IQ studies published between 1988-2012 in a meta-analysis. The consistent results found lower IQ in the “high-fluoride” villages compared with the low-fluoride villages, averaging 7 IQ points lower. Most were at levels above 0.7 mg/l. However, water concentration is a poor assessment of individual fluoride exposure because individual water consumption varies significantly.^5^

INCREASED IQ

In 2022, [Ibarluzea](https://www.sciencedirect.com/science/article/pii/S0013935121014821) reported results in conflict with previous human studies. Over 90% of the human studies reported statistically significant adverse effects and a few reported no effect. The Ibarluzea study is singular in that boys had up to an apparent 28 IQ increase (GCI or General Cognitive Index see Supplement Table 2 in Ibarluzea’s supplement) in the non-fluoridated zone per 1 mg/g of mother’s MUFcr. Boys IQ at age 1 were normal but age 4 showed the significant increased IQ. Girls did not show a significant change. Lack of effect at age 1 and a delayed IQ increase three years later does not appear to fit with known effects of developmental neurotoxins and the study is an outlier with inconsistencies.

Considering the bell curve, IQ moving a couple standard deviations, would result in a clear advantage in schools and work for males on CWF. However, the significant benefit is not obvious in the public at large.

The Ibarluzea study references a study by Xu as precedent, which also reported benefit up to 1.7 mg/L. The Xu study has been retracted, leaving the Ibarluzea study as singular in reporting benefit.

All scientific knowledge both observational and experimental is incomplete and should be in a state of continual discovery. CWF is not an exception. Public health policy decisions on acceptable levels of risk require judgment based on the underlying science, ethics, laws and all streams of evidence.

Income benefit from higher IQ may have more to do with personality, opportunity and ambition and IQ does not appear to show a likelihood of higher total wealth, in part because those with higher IQ also get into financial difficulty. However, IQ does have an effect on groups of people than a predictor of individual income.

**SUPPLEMENT E:** Tables 1 and 2.

ASSUMPTIONS

| ASSUMPTIONS INCLUDE: |
| --- |
| “Steady state” of exposures and outcomes |
| 1.46% of the population is at each year of age |
| Dental fluorosis and reduce IQ are the only adverse health effects |
| All treatment is considered successful and no compensation for pain, suffering, time or travel |
| Published capital, operating costs and benefits reported by Ko are used |
| Compensation for high quality dental fluorosis treatment based on patient perception |
| Cost of treatment based on 2022 USA insurance and cosmetic fees |
| CWF is 0.7 mg/L fluoride and non-fluoridated at <0.1 mg/L fluoride |
| CWF contributes 80% of individual excess exposure |
| “Harm is the cost not the repairs” |
| General inflation of 3.57% and dental inflation 4.33% |
| 25% of Infants exclusively breast fed at six months |
| All dollars are converted to 2022 dollars. |
| IQ is used as a measurement of cognitive development |
| IQ is one of several correlates to higher earnings |

Costs for 4 retreatments are provided in Tables 1 and 2.

Table 1

| Option A: Micro-abrasion bleach and minor restorations every 12 years | Compensation available | Less cost of treatment. (Re-treatment inflation at 4.33% annually.) | Balance is then invested at 3.57% compounded annually |
| --- | --- | --- | --- |
| #1 | $7,200 - | $1,200 = | $6,000 @ 3.57% compounded 10 years = $9,140 |
| #2 | $9,140 - | $1,995 = | $7,144 @ 3.57% compounded 10 years =$10,884 |
| #3 | $10,884 - | $3,319 = | $7,565 @ 3.57% compounded 10 years =$11,524 |
| #4 | $11,524 - | $5,519 = | $6,004 @ 3.57% compounded 10 years =$9,147 |
| #5 | $9,147 - | $9,179 = | ($32) |

Table 2

| Option B Comprehensive Treatment | Compensation | Less cost of treatment. (Re-treatment inflation at 4.33% annually) | Balance is then invested at 3.57% compounded annually |
| --- | --- | --- | --- |
| #1 | $72,000 - | $12,000 = | $60,000 @ 3.57% compounded 10 years =$91,403 |
| #2 | $91,403- | $19,957= | $71,446 @ 3.57% compounded 10 years =$108,839 |
| #3 | $108,839- | $33,190= | $75,649 @ 3.57% compounded 10 years =$115,242 |
| #4 | $115,242- | $55,197= | $60,045 @ 3.57% compounded 10 years =$91,471 |
| #5 | $91,471- | $91,796= | ($325) |

See [Dental services price inflation, 1935→2024 (in2013dollars.com)](https://www.in2013dollars.com/Dental-services/price-inflation)

**SUPPLEMENT F:** NTP (National Toxicology Program) Board of Scientific Counselors Working Group Report on the Draft State of the Science Monograph and the Draft Meta-Analysis Manuscript on Fluoride [Report.](https://ntp.niehs.nih.gov/sites/default/files/2023-05/BSC_WG_Report_Final_Version_BSC_approved051623_508.pdf) Monograph 08, May 2022

The USA Health and Human Services has, to date, blocked the publishing of the scientific report which has taken over eight years, several peer reviews, and a court order to release a draft of the document. Evidence is presented in an interview: [Fluoride On Trial: The Censored Science on Fluoride and Your Health | Childrens Health Defense](https://live.childrenshealthdefense.org/chd-tv/events/fluoride-on-trial-the-censored-science-on-fluoride-and-your-health/fluoride-on-trial/)

When Fluoride’s potential developmental neurotoxicity was nominated in 2015 for NTP review, the NTP Board of Scientific Counselors agreed and we were told by the Director it would take perhaps 2 years for the final report.

As of June, 2024, over eight years after NTP started, the final report on fluoride’s developmental neurotoxicity has not been published because (based on FOI documents) the scientific evidence was quashed and the scientific integrity compromised by the Assistant Secretary for Health.

Anyone evaluating fluoridation’s benefit and risks is advised to watch the interview of Michael Connett JD by Mary Holland, JD. The interview presents statements under oath from the Director of CDC’s Oral Health Division, Casey Hannan, EPA’s Representative Dr. Edward Ohanian, EPA’s Neurotoxicologist, Dr. Stanley Barone, Jr., and EPA Office of Water, Joyce Donohue. Experts such as head of the NTP Brian Berridge, Former Director of NIEHS and NTP Linda Birnbaum, and expert scientists on fluoride are presented.

This interview will give greater context and documentation to understand current USA Federal inaction on protecting the public from Fluoride’s harm to the developing brain.

**POLITICIANS QUASHED SCIENCE**

Court Declaration by Dr. Linda Birnbaum, former Director of NIEHS and NTP

*“As someone who believes deeply in NTP’s science-based mission, I am concerned by the recent course of events with the fluoride monograph. The decision to set aside the results of an external peer review process based on concerns expressed by agencies with strong policy interests on fluoride suggests the presence of political interference in what should be a strictly scientific endeavor.”*

Dr. Wolf at NTP/NIH/NIEHS in April 28, 2022, emailed (FOI document) to CDC Casey Hannon and others that the scientists presenting the draft to their superiors at HHS, considered the analysis and conclusions were set, *“We are sharing this document for your awareness. At this time the analysis and the conclusions are set.”*

Over 500 studies were evaluated and *“Seventy-two studies assessed association between fluoride exposure and IQ in children.”*

The Dental lobby (ASTDD a private company funded by CDC) took steps to change the conclusion from “presumed” to “moderate confidence” of fluoride’s developmental neurotoxicity.

When asked by the Court what more information would be needed for this EPA expert to change their opinion that Fluoridation is safe, responded. “one or two more studies” are needed.

The draft is over 700 pages. The meta-analysis certainly supports the original conclusion that Fluoride is presumed to be a developmental neurotoxin, even at Fluoridation concentrations.
